# Blood‐based genes and co‐expression network levels are associated with AD/MCI diagnosis, cognitive, and neuroimaging phenotypes and preserved in the brain

**DOI:** 10.1002/alz70862_109896

**Published:** 2025-12-23

**Authors:** Xuan Chen, Xue Wang, Joseph S. Reddy, Zachary Quicksall, Thuy T. Nguyen, Denise A. Reyes, Val J Lowe, Ronald Petersen, Kejal Kantarci, Kwangsik Nho, Mariet Allen, Minerva M. Carrasquillo, Andrew J. Saykin, Nilüfer Ertekin‐Taner

**Affiliations:** ^1^ Mayo Clinic, Jacksonville, FL USA; ^2^ Mayo Clinic, Rochester, MN USA; ^3^ Department of Neurology, Mayo Clinic, Rochester, MN USA; ^4^ Department of Radiology, Mayo Clinic, Rochester, MN USA; ^5^ Center for Neuroimaging, Department of Radiology and Imaging Sciences, Indiana University School of Medicine, Indianapolis, IN USA; ^6^ Mayo Clinic in Florida, Jacksonville, FL USA; ^7^ Center for Neuroimaging, Indiana University School of Medicine, Indianapolis, IN USA

## Abstract

**Background:**

Most existing biomarkers for Alzheimer’s disease (AD) inform on core AD pathologies in the central nervous system, namely amyloid, tau and neurodegeneration (ATN). However, molecular disease mechanisms underlying AD are complex and heterogeneous, highlighting the need for cost‐effective blood‐based biomarkers that reflect brain molecular changes in AD beyond ATN.

**Method:**

Using peripheral blood transcriptome data from the Mayo Clinic Study of Aging(MCSA) and the Alzheimer's Disease Neuroimaging Initiative, we identified genes associated with AD or mild cognitive impairment (MCI) diagnosis, cognitive, and harmonized neuroimaging measures in two cohorts, followed by meta‐analysis. We identified consensus gene co‐expression modules in MCSA and ADNI using WGCNA and assessed the association between module eigengenes and aforementioned measures. Using seven bulk brain RNAseq cohorts, we confirmed the preservation of coexpression modules across blood and brain. Enrichment analyses of Gene Ontology (GO) terms and cell‐types were performed using significant and/or hub genes. The MCSA transcriptome was further deconvoluted to obtain cell‐type proportions and cell‐type specific expression.

**Result:**

Blood transcriptome modules M5 and M8 are upregulated in AD/MCI and associate with worse cognition, whereas M1, M10, M13, M16, and M21 are downregulated in AD/MCI and associate with better cognition. M17 associates with more microhemorrhages. Cell‐type enrichment analysis revealed that M5 is enriched in basophils, monocytes, and neutrophils; M8 in monocytes and neutrophils; M13 in natural kills cells; M17 in megakaryocytes; M21 in B cells. GO analysis shows broad consistency with the cell‐type enrichment analysis. M1, 5, and 8 are preserved in the brain network modules. From these modules, we prioritized transcripts significantly associated with at least one AD‐related phenotype in both blood and brain. Cell‐type deconvolution analysis shows that prioritized transcripts in M1 associate with better cognition and higher metabolic activity in B cells.

**Conclusion:**

We identified blood transcripts and modules associated with AD/MCI, cognition, or neuroimaging, with three preserved in brain networks. These three modules represent centrally linked peripheral molecular signatures that can reveal perturbed brain pathways and cell level transcriptional changes in AD/MCI. We aim to further investigate the prioritized transcripts in additional datasets to establish them as potential novel biomarkers and therapeutic targets.

